# The different predictive value of mean platelet volume-to-lymphocyte ratio for postoperative recurrence between non-muscular invasive bladder cancer patients treated with intravesical chemotherapy and intravesical chemohyperthermia

**DOI:** 10.3389/fonc.2022.1101830

**Published:** 2023-01-11

**Authors:** Chengbo Wang, Wenjun Jin, Xiaodong Ma, Zhilong Dong

**Affiliations:** ^1^ The Department of Urology, Lanzhou University Second Hospital, Lanzhou University, Lanzhou, Gansu, China; ^2^ The Department of Urology, Wuwei Cancer Hospital of Gansu Province, Wuwei, Gansu, China

**Keywords:** hyperthermia, intravesical chemotherapy, mean platelet volume to lymphocyte ratio, non-muscular invasive bladder cancer, platelet to lymphocyte ratio, recurrence, systemic immune inflammation index

## Abstract

**Introduction:**

The inflammatory response plays a potential role in postoperative recurrence in patients with non-muscular invasive bladder cancer (NMIBC). We aimed to investigate whether platelet-to-lymphocyte ratio (PLR), mean platelet volume to lymphocyte ratio (MPVLR), and the systemic immune-inflammatory index (SII) have prognostic values in NMIBC treated with conventional intravesical chemotherapy or intravesical Chemohyperthermia (CHT) and the differences between them.

**Materials and methods:**

A retrospective cohort study was conducted on 222 patients with NMIBC treated with Intravesical Chemotherapy or Intravesical CHT between January 2016 and December 2020. Within a week before surgery, PLR, MPVLR, and SII were determined based on routine blood settling. The optimal cutoff value of each index was determined using the receiver operating characteristic curve, and various groups were categorized accordingly. The factors influencing the prognosis of NMIBC patients receiving various treatments were investigated using the Kaplan- Meier survival curve and the Cox regression model.

**Results:**

69 cases (46.3%) in the gemcitabine (GEM) group had tumor recurrence and 19 (12.8%) of them progressed to muscle-invasive bladder cancer (MIBC) or got metastasis, while 19 cases (26.0%) in the CHT group recurred and 2 (2.7%) progressed. Elevated PLR, MPVLR, and SII were associated with higher recurrence rates in the GEM group. Meanwhile, PLR and MPVLR were the independent risk factors. While in the CHT group, high PLR and SII were related to postoperative recurrence and none of them were independent risk factors.

**Conclusion:**

The preoperative clinical inflammatory indexes PLR, SII, and MPVLR have certain predictive value for the postoperative recurrence-free survival (RFS) in NMIBC patients treated with intravesical chemotherapy while PLR and SII can predict the prognosis of NMIBC patients treated with intravesical CHT, which indicates that intravesical CHT may stop tumor recurrence by influencing the effect of mean platelet volume on tumor growth through some unknown mechanisms.

## 1 Introduction

Bladder cancer (BC) is one of the most common cancers in the urinary system, and its incidence ranks eleventh in the global incidence of malignant tumors (1). 70% to 80% of patients with BC are diagnosed with non-muscle invasive bladder cancer (NMIBC) (2). The standard treatment for NMIBC is getting the transurethral resection of bladder tumor (TURBT) with postoperative intravesical chemotherapy or Bacillus Calmette-Guerin (BCG) infusion, and then undergoing a second surgery when it is necessary. However, the recurrence rate of NMIBC after surgery has remained high. Chemohyperthermia (CHT) is a new method for postoperative perfusion, which increases the absorption of chemotherapy drugs in the bladder wall by locally heating the bladder to improve its efficacy. Many studies have shown that its efficacy in NMIBC is better than intravesical chemotherapy and it is certainly safe. Using biomarkers to predict the prognosis of NMIBC can benefit the treatment of NMIBC and reduce its recurrence and progression.

Lymphocytes are involved in cytotoxic cell death and inhibit tumor cell proliferation and metastasis. Platelets are involved in different stages of tumor angiogenesis, including proliferation, migration, extracellular matrix degradation, and adhesion of endothelial cells. Platelet-to-lymphocyte ratio (PLR) has been shown to be significantly associated with cancer-specific survival (CSS) and overall survival (OS) in urothelial carcinoma patients who underwent total cystectomy (3). Mean platelet volume (MPV), a known biomarker of pro-inflammatory and prothrombotic states, is considered an inflammatory marker of cardiovascular, rheumatic, and digestive diseases. There are few studies on MPV to lymphocyte ratio (MVPLR) for tumor prognosis. In addition, there is little research on the relationship between the systemic immune-inflammatory index (SII) and the prognosis of NMIBC patients. But SII has a good prognostic value for patients with hepatocellular carcinoma and renal clear cell carcinoma.

The purpose of this study was to investigate whether PLR, MPVLR, and SII have prognostic value in NMIBC treated with conventional intravesical instillation or intravesical CHT. Will they play different prognostic roles due to different ways of perfusion?

## 2 Materials and methods

### 2.1 Study design and data collection

The participants were composed of patients with NMIBC who underwent the first transurethral bladder tumor resection (TURBT) at Lanzhou University Second Hospital and Gansu Wuwei tumor Hospital between January 2016 and December 2020. Exclusion criteria were as follows:①Preoperative urinary tract infection or systemic infection, serious complications (such as bladder perforation, severe bleeding) occurred during or after surgery;②Patients with immune system diseases, blood diseases or other malignant tumors;③Neurogenic bladder;④Due to various reasons (psychological disorders, urethral stricture, etc.), the chemotherapy sessions were not completed according to the plan;⑤Lost to follow-up due to various reasons. Immediately after receiving TURBT (within 48h), the patients at Lanzhou University Second Hospital (GEM group) were treated with gemcitabine (GEM) for 8 weekly induction sessions and then 10 monthly maintenance sessions. Meanwhile, the patients at Gansu Wuwei tumor Hospital (CHT group) were treated with intravesical CHT with GEM/THP-adriamycin (THP) 3 times right after taking the operation. These three sessions were conducted 48h apart, and then the patients took monthly subsequent treatments of common intravesical chemotherapy for the next year. We used the BR-TRG-I body cavity hyperthermia therapeutic instrument (developed in China) for intravesical chemohyperthermia. The water inlet of the three-chamber catheter is connected to the perfusion tube of the instrument, and the liquid from the water outlet flows back into the instrument to form a circulatory system so that the heated drugs can be injected into the bladder at a constant temperature and circularly. The speed of perfusion is about 150ml/min, the temperature difference is less than 1°C, and the duration is 45min.

From computerized medical records and telephone consultations, the participants’ clinical information, including age, gender, tumor number, tumor size, tumor pathological grade, and tumor pathological stage, were gathered. After taking the operation, the patients received a cystoscopy examination 3 months later and then underwent a cystoscopy examination every six months. Urine cytology was performed every 3 months, and a color Doppler ultrasound or CT examination of the urinary system was performed every six months. The primary index of survival analysis was recurrence-free survival (RFS). RFS data were mostly collected by outpatient review or telephone follow-up. RFS was calculated as the interval between the initial pathological confirmation of NMIBC and the initial postoperative recurrence. Follow-ups were required by May 1, 2022.

The neutrophil count, lymphocyte count, platelet count, and MPV from the patient’s blood routine test results within one week prior to operation TURBT were used to determine PLR, MPVLR, and SII. PLR was calculated by dividing absolute platelet counts by absolute lymphocyte counts. MPVLR was computed by dividing the MPV by absolute lymphocyte counts. SII was calculated by the product of neutrophil counts and platelet counts to lymphocyte counts. PLR, MPVLR, and SII were categorized into two groups according to different optimal cut-off values for each outcome. The receiver operating characteristic curve (ROC curve), based on the method of the Youden index, was used to calculate the optimal cut-off values of them. These cut-off points optimized the differentiating functionality of the PLR, MPVLR, and SII when an equal weight was given to the specificity and sensitivity (4, 5).

### 2.2 Statistical analyses

All statistical analyses have been carried out by the usage of SPSS 26.0 version statistical software (IBM) and GraphPad Prism version 7.0 (GraphPad Software Inc.). We chose recurrence as the primary outcome and calculated the best cut-off value of each index with the ROC curves of PLR, MPVLR and SII combined with the maximum Youden index. Then we classified these patients into a high-count group and a low-count group accordingly. The clinical data between two groups (high or low) of the same variable were compared. Counting variables were compared by using the X**^2^** test or Fisher exact probability method. Kaplan- Meier method was used to draw survival curves. We also used the Log-rank test to compare the RFS among different PLR, MPVLR, and SII groups. The risk factors affecting prognosis were analyzed by the Cox regression model.

All P values were two-sided, and a value of less than 0.05 was considered significant. The study protocols were reviewed and approved by the Medical Ethics Committee of Lanzhou University Second Hospital (File No. 2022A-299) in June 2022.

## 3 Results

A total of 222 cases were enrolled in this study, which consisted of 149 patients in the GEM group and 73 patients in the CHT group. The median follow-up time was 42 months (interquartile range:17-48 months). 69 cases in the GEM group had tumor recurrence and 19 of them progressed to MIBC or got metastasis, 3 of them died of cancer. while 19 cases in the CHT group recurred and 2 progressed, none of them died of cancer.

### 3.1 Efficacy comparison between the GEM group and the CHT group

**Table 1** shows the clinical baseline comparison between the GEM group and the CHT group. The patient’s gender (X**^2^** =0.200, p=0.655), age (t=0.709, p=0.479), tumor size (t=-1.365, p = 0.147), number of tumors (t=1.223, p=0.223), tumor pathological grade (X**^2^** =0.348, p=0.555) and pathological stage (X**^2^** =0.436, p=0.509) all confirmed no significant difference between the two groups (p>0.05).

**Table 1:** Clinicopathological data of patients with NMIBC between the GEM group and the CHT group.

Clinicopathological variable	CHT group (n = 73)	GEM group (n = 149)	t/X²	P
**Gender**			0.200	0.655
Male	57	123		
Female	16	26		
**Preoperative age**			0.709	0.479
≤60yr.	37	69		
60-70yr.	25	48		
≥70yr.	11	32		
**Tumour size**			-1.365	0.147
≤3cm	62	109		
>3cm	11	40		
**Amount of tumours**			1.223	0.223
n ≤ 3	57	135		
n>3	16	14		
**Grade**			0.348	0.555
Low	49	94		
High	24	55		
**T stage**			0.436	0.509
Ta	61	119		
T1	12	30		

CHT, Chemohyperthermia; GEM, gemcitabine.

**Figure 1:**
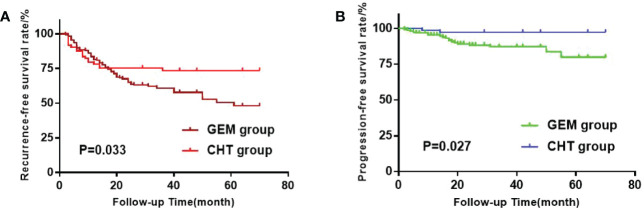
**(A)** Kaplan-Meier survival estimation of the gemcitabine (GEM) group and Chemohyperthermia (CHT) group when recurrence-free survival (RFS) as the outcome. **(B)** Kaplan-Meier survival estimation of the GEM group and CHT group when progression- free survival (PFS) as the outcome.

The survival curves were finished by using The Kaplan-Meier method (**Figures 1A, B**). We used the Log-rank test to compare the RFS and progression-free survival (PFS) between the two groups. The RFS of the GEM group and the CHT group were 43.9% and 26.0%, respectively, which showed a significant difference between them(p=0.033). The PFS rates of the GEM group and the CHT group were 12.1% and 2.7%, respectively, which also showed a significant difference (p=0.027).

### 3.2 AUC analyses of PLR, MPVLR, and SII

**Figure 2:**
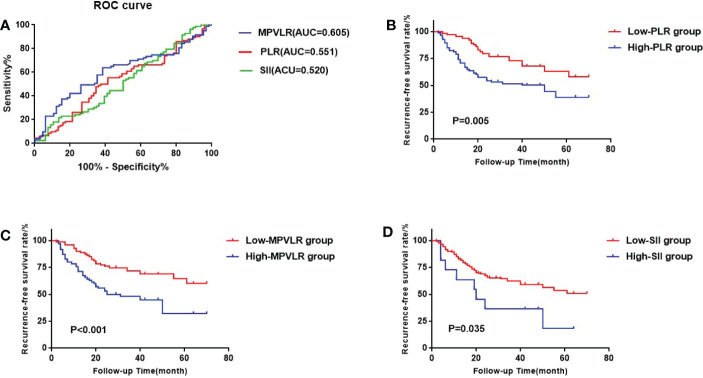
**(A)** the receiver operating characteristic (ROC) curves of the best cutoff values of platelet-to-lymphocyte ratio (PLR) PLR, mean platelet volume to lymphocyte ratio (MPVLR), and systemic immune inflammatory index (SII) of the GEM group. **(B)** Comparison of sur-vival curves between the high PLR group and the low PLR group. **(C)** Comparison of survival curves between the high MPVLR group and the low MPVLR group. **(D)** Comparison of survival curves between the high SII group and the low SII group.

In the GEM group, The areas under the ROC curves of PLR, MPVLR, and SII were 0.551, 0.605, and 0.520, respectively. The optimal cut-off values were figured out by choosing the maximum value of the Youden Index as follows: The cut-off value of PLR, MPVLR, and SII were 93.075(sensitivity 0.667 and specificity 0.506), 6.377(sensitivity 0.621 and specificity 0.639), and 707.385 (sensitivity 0.121 and specificity 0.964), respectively. The PLR was classified into the low PLR group (64 cases) and the high PLR group (85 cases) based on 93.075. The MPVLR was classified into the low MPVLR group (79 cases) and the high MPVLR group (70 cases) based on 6.377. The SII was classified into the low SII group (138 cases) and the high SII group (11 cases) based on 707.385(**Figure 2A**).

**Figure 3:**
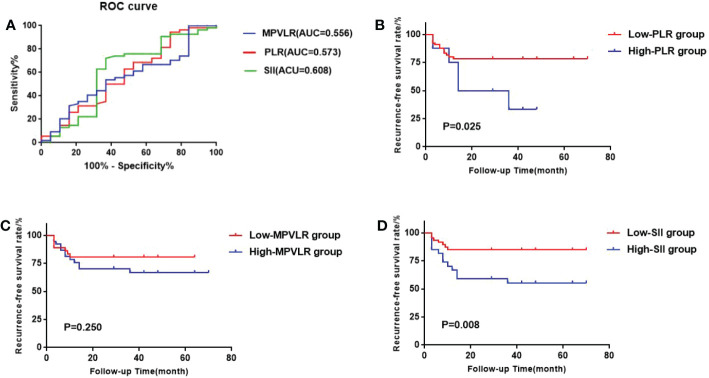
**(A)** ROC curves of the best cutoff values of PLR, MPVLR, and SII of the CHT group. **(B)** Comparison of survival curves between the high PLR group and the low PLR group. **(C)** Comparison of survival curves between the high MPVLR group and the low MPVLR group. **(D)** Comparison of survival curves between the high SII group and the low SII group.

In the CHT group, The areas under the ROC curves of PLR, MPVLR, and SII were 0.573, 0.556, and 0.608, respectively. The optimal cut-off values were selected in an identical way to the GEM group: the PLR cut-off value was 183.863(sensitivity 0.263 and specificity 0.944); the MPVLR cut-off value was 7.267(sensitivity 0.632 and specificity 0.537); the SII cut-off value was 575.335 (sensitivity 0.632 and specificity 0.722). The PLR was classified into the low PLR group (65 cases) and the high PLR group (8 cases) on the basis of 183.863. The MPVLR was classified into the low MPVLR group (36 cases) and the high MPVLR group (37 cases) on the basis of 7.267. The SII was classified into the low SII group (57 cases) and the high SII group (16 cases) on the basis of 575.335 (**Figure 3A**).

### 3.3 The relationship between patient clinical data and PLR, MPVLR and SII

In the GEM group, as **Table 2** shows, the patient’s gender, age, tumor size, number of tumors, tumor pathological grade, and pathological stage did not differ significantly across groups. In the CHT group, shown in **Table 3**, only tumor size between the high PLR group and the low PLR group showed significant differences (X**^2 =^** 5.775, p=0.016). Other clinical features also did not differ significantly across the groups.

**Table 2:** Correlation between clinical data of the GEM group and PLR, SII, and MPVLR.

Variables	High-MPVLR group (n = 70)	Low-MPVLR group (n = 79)			High-PLR group (n = 85)	Low-PLR group (n = 64)			High-SII group (n = 11)	Low-SII group (n = 138)		
	n(%)	n(%)	t/X²	P	n(%)	n(%)	t/X²	P	n(%)	n(%)	t/X²	P
**Gender**			0.009	0.926			0.259	0.611			2.950	0.086
Male	58 (82.6)	65 (82.3)			69 (81.2)	54 (84.4)			7 (63.6)	116 (84.1)		
Female	12 (17.4)	14 (17.7)			16 (18.8)	10 (15.6)			4 (36.4)	22 (15.9)		
**Preoperative age**			0.842	0.656			2.470	0.291			0.278	0.870
≤60yr.	30 (42.8)	39 (49.4)			36 (42.4)	33 (51.6)			5 (45.4)	64 (46.4)		
60-70yr.	23 (32.9)	25 (31.6)			27 (31.8)	21 (32.8)			3 (27.3)	45 (32.6)		
≥70yr.	17 (24.3)	15 (20.0)			22 (25.8)	10 (15.6)			3 (27.3)	29 (21.0)		
**Tumour size**			0.441	0.507			1.108	0.292			0.454	0.500
≤3cm	53 (75.7)	56 (70.1)			65 (76.5)	44 (68.8)			9 (81.8)	100 (72.5)		
>3cm	17 (24.3)	23 (29.9)			20 (23.5)	20 (31.2)			2 (18.2)	38 (27.5)		
**Amount of tumours**			0.057	0.812			0.313	0.576			1.077	0.299
n ≤ 3	63 (90.0)	72 (91.1)			78 (91.8)	57 (89.1)			9 (81.8)	126 (91.3)		
n>3	7 (10.0)	7 (8.9)			7 (8.2)	7 (10.9)			2 (18.2)	12 (8.7)		
**Grade**			1.156	0.282			0.664	0.415			0.002	0.969
Low	41 (58.6)	53 (67.1)			56 (65.9)	38 (59.4)			7 (63.6)	87 (63.0)		
High	29 (41.4)	26 (32.9)			29 (34.1)	26 (40.6)			4 (36.4)	51 (37.0)		
**T stage**			0.201	0.654			0.211	0.646			0.901	0.343
Ta	57 (81.4)	62 (78.5)			69 (81.2)	50 (78.1)			10 (90.1)	109 (79.0)		
T1	13 (18.6)	17 (21.5)			16 (18.8)	14 (21.9)			1 (9.9)	29 (21.0)		

GEM, gemcitabine;PLR, the ratio of platelets to lymphocytes; SII, systemic immune inflammation index; MPVLR, the ratio of mean platelet volume to lymphocytes.

**Table 3:** Correlation between clinical data of the CHT group and PLR, SII, and MPVLR.

Variables	High-MPVLR group (n = 16)	Low-MPVLR group (n = 57)			High-PLR group (n = 8)	Low-PLR group (n = 65)			High-SII group (n = 27)	Low-SII group (n = 46)		
	n(%)	n(%)	t/X²	P	n(%)	n(%)	t/X²	P	n(%)	n(%)	t/X²	P
**Gender**			0.114	0.736			1.275	0.259			1.489	0.222
Male	12 (75.0)	45 (78.9)			5 (62.5)	52 (80.0)			19 (70.4)	38 (82.6)		
Female	4 (25.0)	7 (21.1)			3 (37.5)	13 (20.0)			8 (29.6)	8 (17.4)		
**Preoperative age**			0.868	0.648			0.999	0.607			2.333	0.311
≤60yr.	6 (37.5)	31 (54.4)			3 (37.5)	34 (52.3)			14 (51.9)	23 (50.0)		
60-70yr.	6 (37.5)	19 (33.3)			4 (50.0)	21 (32.3)			7 (10.9)	18 (39.1)		
≥70yr.	4 (25.0)	7 (12.3)			1 (12.5)	10 (15.4)			6 (22.2)	5 (10.9)		
**Tumour size**			0.217	0.641			5.775	0.016			2.715	0.099
≤3cm	13 (81.3)	49 (86.0)			4 (50.0)	58 (89.2)			20 (74.1)	42 (91.3)		
>3cm	3 (18.7)	8 (14.0)			4 (50.0)	7 (10.8)			7 (25.9)	4 (8.7)		
**Amount of tumours**			1.984	0.159			2.461	0.117			2.476	0.116
n ≤ 3	13 (81.3)	53 (93.0)			6 (75.0)	60 (92.3)			22 (81.5)	44 (95.7)		
n>3	3 (18.7)	4 (7.0)			2 (25.0)	5 (7.7)			5 (18.5)	2 (4.3)		
**Grade**			0.198	0.656			0.253	0.615			2.598	0.107
Low	10 (62.5)	39 (68.4)			6 (75.0)	43 (66.2)			15 (55.6)	34 (73.9)		
High	6 (37.5)	18 (31.6)			2 (25.0)	22 (33.8)			12 (44.4)	12 (26.1)		
**T stage**			0.231	0.630			2.901	0.088			0.135	0.713
Ta	14 (87.5)	47 (82.5)			5 (62.5)	56 (86.2)			22 (81.5)	39 (84.8)		
T1	2 (12.5)	10 (17.5)			3 (37.5)	9 (13.8)			5 (18.5)	7 (15.2)		

CHT, Chemohyperthermia; PLR, the ratio of platelets to lymphocytes; SII, systemic immune inflammation index; MPVLR, the ratio of mean platelet volume to lymphocytes.

### 3.4 Survival analyses of the GEM group and the CHT group

We used the Kaplan-Meier method to form the survival curves while the log-rank test was used to compare the RFS among the different PLR, MPVLR, and SII groups. In the GEM group (**Figures 2B-D**), The RFS of the high PLR group and the low PLR group were 51.8% and 34.4%. The difference between these two groups was significant(p=0.005). The RFS of the high MPVLR group and the low MPVLR group were 57.1% and 32.9%, which showed a significant difference between them (p<0.001). The RFS of the high SII group and the low SII group were 72.8% and 42.0%. There was also a significant difference between them (p=0.035). In the CHT group (**Figures 3B-D**), with significant differences, the RFS of the high PLR group and the low PLR group were 62.5% and 21.5% (p=0.025), the RFS of the high SII group and the low SII group were 44.4% and 15.2%(p=0.008). However, the RFS of the high MPVLR group and the low MPVLR group were 32.4% and 19.4%, with no significant difference (p=0.250).

The univariate and multivariate analyses were going with the Cox proportional hazard risk regression model. **Table 4** shows that in the GEM group, the univariate analysis indicated that males (HR=1.822, p=0.032), 60-70yr. (HR=1.911, p=0.024), high tumor pathological grade (HR=2.237, p<0.001), high MPVLR (HR=2.280, p<0.001), high PLR (HR=2.010, p=0.008) and high SII (HR=2.160, p=0.041) were statistically significant covariates of tumor recurrence. The significant indexes of univariate analysis have been analyzed with the aid of multivariate analysis and the consequences confirmed that high tumor pathological grade (HR=0.403, p<0.001), high MPVLR (HR=2.042, p=0.007) and high PLR (HR=2.119, p=0.008) were considerable variables of tumor recurrence in the NMIBC patients treated with intravesical GEM. **Table 5** reveals that in the CHT group, the univariate analysis indicated tumor size>3cm (HR=3.343, p=0.015), amount of tumors>3(HR=3.202, p=0.039), T1 stage (HR=3.599. p=0.007), high PLR (HR=2.984, p=0.036) and high SII (HR=3.232, p=0.014) were statistically significant covariates of tumor recurrence. The multivariate analysis showed that tumor size>3cm (HR=0.307, p=0.039) and T1 stage (HR=0.247. p=0.011) were considerable variables of tumor recurrence in the NMIBC patients treated with intravesical CHT.

**Table 4:** Univariate and multivariate analysis with a Cox proportional hazard model of risk factors on RFS of the patients treated with intravesical GEM.

Variables	Univariate analysis			Multivariate analysis		
	HR	95% CI	P-value	HR	95% CI	P-value
Gender						
Female	1.822	1.053-3.151	0.032	0.591	0.324-1.077	0.086
Preoperative age(≤60yr.)						
60-70yr.	1.911	1.089-3.352	0.024	0.633	0.381-1.053	0.078
≥70yr.	0.646	0.244-1.707	0.378	—	—	—
Tumour size(≤3cm)						
>3cm	1.587	0.961-2.619	0.071	—	—	—
Amount of tumours(n ≤ 3)						
n>3	1.670	0.828-3.369	0.152	—	—	—
Grade(Low)						
High	2.237	1.390-3.601	<0.001	0.403	0.241-0.673	<0.001
T stage(Ta)						
T1	1.159	0.653-2.058	0.613	—	—	—
MVPLR(Low)						
High	2.280	1.388-3.745	<0.001	2.042	1.211-3.443	0.007
PLR(Low)						
High	2.010	1.202-3.359	0.008	2.119	1.216-3.695	0.008
SII(Low)						
High	2.160	1.030-4.529	0.041	0.915	0.395-2.116	0.835

GEM, gemcitabine; CI, confidential interval; HR, hazard ratio; PLR, the ratio of platelets to lymphocytes; SII, systemic immune inflammation index; MPVLR, the ratio of mean platelet volume to lymphocytes.

**Table 5:** Univariate and multivariate analysis with a Cox proportional hazard model of risk factors on RFS of the patients treated with intravesical CHT.

Variables	Univariate analysis			Multivariate analysis		
	HR	95% CI	P-value	HR	95% CI	P-value
Gender						
Female	1.732	0.658-4.559	0.266	—	—	—
Preoperative age(≤60yr.)						
60-70yr.	1.556	0.584-4.146	0.377	—	—	—
≥70yr.	1.181	0.608-2.293	0.624	—	—	—
Tumour size(≤3cm)						
>3cm	3.343	1.266-8.827	0.015	0.307	0.100-0.942	0.039
Amount of tumours(n ≤ 3)						
n>3	3.202	1.059-9.677	0.039	0.758	0.225-2.549	0.654
Grade(Low)						
High	1.672	0.671-4.162	0.269	—	—	—
T stage(Ta)						
T1	3.599	1.413-9.163	0.007	0.247	0.084-0.725	0.011
MVPLR(Low)						
High	0.612	0.178-2.102	0.436	—	—	—
PLR(Low)						
High	2.984	1.074-8.293	0.036	1.253	0.364-4.312	0.721
SII(Low)						
High	3.232	1.271-8.214	0.014	0.398	0.137-1.161	0.092

CHT, Chemohyperthermia; CI, confidential interval; HR, hazard ratio; PLR, the ratio of platelets to lymphocytes; SII, systemic immune inflammation index; MPVLR, the ratio of mean platelet volume to lymphocytes.

## 4 Discussion

There have been nearly 20 years of research on intravesical CHT. Most of the studies have shown that the efficacy of intravesical CHT for NMIBC is better than traditional intravesical chemotherapy. In the research of Arends et al., 190 NMIBC patients were randomized in that controlled, open-label, multicentre trial for 1-yr CHT and 1-yr BCG immunotherapy. They found that the 24-mo RFS in the PP analysis was 81.8% in the CHT group compared with 64.8% in the BCG group (p=0.02) and turned out that CHT is a safe and effective treatment option in patients with intermediate- and high-risk papillary NMIBC (6). Our retrospective cohort study figured out a similar result that CHT can reduce the recurrence rate of NMIBC and also prevent tumor progression.

The complex connection between tumor cells and the body’s immune system influences tumor initiation and progression (7, 8). Neutrophilia can induce DNA damage by secreting reactive oxygen species, stimulating cancer cell proliferation, and promoting new blood vessels around cancer tissues (9). Studies have shown that neutrophils migrate to peritumoral tissues to participate in the induction of hypoxia and the release of vascular endothelial growth factor (VEGF) to promote tumor growth (10, 11). The progression of hypoxic necrosis within the tumor also further induces an innate immune response, resulting in an overall rise in neutrophil counts (12, 13). The increasing evidence suggests that the causal relationship between local immune responses and systemic inflammation in many malignancies is not an accident (14). Lymphocytes are considered as a basal anti-tumor defense line (15). Sharma et al. revealed that a greater number of CD8 + infiltrating lymphocytes improves the prognosis of patients with MIBC (16). Furthermore, advanced bladder cancer is associated with unstable host defense and a decreased number of lymphocytes (17). Cancer microenvironment cells stimulate monocytes and neutrophils to secrete, inter alia, interleukin 6 (IL-6), VEGF, and transforming growth factor β (TGF-β), causing general immunosuppression by inducing apoptosis of lymphocytes and a decrease in lymphopoiesis (18–21).

In addition, circulating tumor cells (CTCs) are shielded from natural killer cell cytolysis by platelets, which enclose the tumor cells in a thrombus (22). tumor cells stimulate platelets through specific mechanisms to promote a persistent adhesion between them and tumor cells, which increased platelet numbers, led to hypercoagulation, and raised thrombosis risks in cancer patients. Some of the chemicals that platelets emit have the ability to increase the proliferation and development of tumor cells (23, 24). Moreover, Platelets with elevated MPV have strong reactivity and aggregation ability, so MPV is also considered a surrogate marker of platelet activation. Activated platelets can recruit neutrophils and monocytes and release Chemokines like p-selectin, VEGF, and IL-8, which play a role in promoting hemostasis, vascular remodeling, and inflammation (25, 26). Therefore, we believed that MPV should be related to tumor occurrence, progression, and other tumor behaviors.

The suggested NLR reflects the dynamic balance of the host’s inflammatory response and anti-tumor effect and is based on the neutrophil count and lymphocyte count. The probability of locally progressed disease, future disease recurrence, cancer-specific, and all-cause mortality is dramatically enhanced in patients having RC when their preoperative NLR is elevated (27). NLR≧2.41 may be employed as a standalone predictor of postoperative cancer recurrence in patients with NMIBC, according to Mano et al. (28). Considering that there have been many reports of NLR correlation with NMIBC, we chose other 3 indices that few studies have evaluated a particular outcome in patients with NMIBC: PLR, SII, and MPVLR.

PLR is the ratio of platelets to lymphocytes. Jiwei Huang et al. found that the Preoperative PLR value can be used as a negative independent prognostic factor for survival outcomes in patients with localized upper tract urothelial carcinoma (29). However, no study has investigated the prognostic role of PLR for patients with NMIBC. By using the ROC curve and the approximately identical index, our study found the best cut-off value for PLR: 93.075 in the GEM group and 183.863 in the CHT group. In both the GEM group and the CHT group, the RFS rate of the high PRL group was substantially lower than that of the low PLR group (p<0.05). High PLR appeared to be an independent risk factor for postoperative recurrence in the GEM group and was associated with postoperative recurrence in both groups, according to Cox single and multivariate analyses. We noticed that the cut-off value of the CHT group was different from that of the GEM group. We considered the reason may be that patients from the two hospitals may differ in a local environment, lifestyle, patient inflammatory state and then caused this bias.

A recently proposed prognostic indicator is the Systemic Immune Inflammation Index (SII), which incorporates neutrophil count, lymphocyte count, and platelet count. Patients with liver cancer, lung cancer, and kidney cancer have been proven to have a bad prognosis when this indication is uncommon (30, 31). Based on a study, SII is substantially linked to poorer survival outcomes and unfavorable pathological features in BC patients (32). Lower SII was also seemed as a vital independent predictor of response to intravesical BCG immunotherapy and revealed a preferable prognosis in NMIBC patients (33). Similarly, our study showed the result that NMIBC patients with lower SII had a lower risk of tumor recurrence in both the GEM group and the CHT group, although it could not be used as an independent risk factor for tumor recurrence in NMIBC patients treated with intravesical GEM chemotherapy, nor those treated with intravesical CHT. Additionally, compared to the research above, our study’s cut-off value for SII was somewhat different. Given the patients’ health conditions, the varying tumor tolerance levels of various geographic and racial groupings, and the fact that only two facilities were engaged in this retrospective analysis, there may be some discrepancies resulting in a selectivity bias.

MPVLR is the ratio of mean platelet volume (MPV) to lymphocytes. So far MPVLR has been widely studied in cardiovascular disease, and inflammatory response, but few in tumor prognosis. A recent study proved that high MPVLR (≥3.61) was independently associated with higher long-term overall mortality in nonmetastatic ccRCC patients (34). Our study was the first to investigate whether MPVLR was associated with the recurrence of patients with NMIBC. Did MPVLR play different predictive roles between conventional intravesical chemotherapy and intravesical CHT? It turned out that high MPVLR (≥6.377) was independently associated with lower recurrence-free survival in NMIBC patients treated with intravesical GEM chemotherapy. However, in NMIBC patients treated with intravesical CHT, MPVLR had no predictive value for tumor recurrence. We considered the reason for this phenomenon may be that intravesical CHT could stop tumor recurrence by influencing the effect of mean platelet volume on tumor growth through certain mechanisms so that MPVLR could no longer predict the postoperative recurrence in these patients. But more research needs to be done to prove the possibility of these unknown mechanisms.

Furthermore, the results of Cox regression analysis in this study suggested that postoperative recurrence in patients with NMIBC treated with intravesical GEM chemotherapy was related to gender, preoperative age, tumor pathological grade, and tumor pathological grade was an independent risk factor. In the NMIBC patients treated with intravesical CHT, postoperative recurrence was associated with tumor size, amount of tumors, and tumor pathological stage when tumor pathological stage was an independent risk factor. However, we did not use any prognostic scoring model because of the limit of this retrospective study and the lack of other clinical data.

Novel emerging tools such as circulating cell-free DNA (cfDNA), circulating tumor DNA (ctDNA), Whole exome sequencing (WES), and next-generation sequencing (NGS) based strategies represent an attractive platform for diagnosis, longitudinal monitoring, and prognosis prediction to specifically guide the treatment strategy within different settings (35). However, DNA sequencing tools are time-consuming and expensive. Gene mutation will also bring difficulties to their clinical use. Compared with these tools, although the inflammation, immuno-nutrition-based and other blood-related markers also have limits in clinical use because of the lack of reproducibility and prospective studies, they are easy to get and economical. As long as a scoring system can be built to help reduce bias, these biomarkers will play a key role in evaluating tumor prognosis in the future. Besides the predictive values, our study confirms that intravesical CHT could stop tumor recurrence by influencing the effect of mean platelet volume on tumor growth through certain mechanisms, which needs more research to explore.

This research had several restrictions. Because it was a retrospective cohort analysis, this study was unable to gather and assess data on how PLR, SII, and MPVLR variations affected clinical outcomes throughout a follow-up period. Selection bias was unavoidable as a result of the study’s small sample size and dependence on just two centers. Due to the scarcity of data, we were unable to incorporate some possible confounders in our study, including smoking history, concurrent inflammation, medications consumed, and genetic variables. The EORTC scoring model was developed based on patients receiving several different perfusion regimens and was used to predict recurrence and prognosis in NMIBC patients (internally validated c-index for recurrence and progression, 0.66 and 0.75, respectively) (36). Afterwards, a CUETO score model was developed to predict the prognosis of patients treated with BCG perfusion (the c-index of recurrence and progression were 0.636 and 0.687, respectively) (37). However, there has no such scoring model to predict the prognosis of NMIBC patients treated with intravesical CHT. Therefore, we need to expand the sample size and conduct prospective studies to form a scoring model for them in the future.

## 5 Conclusion

Our study demonstrated that elevated PLR, SII, and MPVLR have a certain predictive effect on the postoperative recurrence of patients with NMIBC who were treated with intravesical GEM chemotherapy, of which PLR and MPVLR were two independent risk factors. While PLR and SII, without MPVLR, were associated with postoperative recurrence of patients with NMIBC who were treated with intravesical CHT, which indicated that intravesical CHT could possibly inhibit tumor recurrence in NMIBC patients by influencing the effect of mean platelet volume on tumor cells. Based on the results of our retrospective cohort study, we recommend exploring this relationship and determining the optimal cut-off values of PLR, SII, and MPVLR for patients with NMIBC treated with conventional intravesical chemotherapy or intravesical CHT using other well-designed datasets.

## Data availability statement

The original contributions presented in the study are included in the article/supplementary material. Further inquiries can be directed to the corresponding author.

## Ethics statement

The studies involving human participants were reviewed and approved by the Medical Ethics Committee of Lanzhou University Second Hospital. Written informed consent for participation was not required for this study in accordance with the national legislation and the institutional requirements.

## Author contributions

CW did the data collection and manuscript writing. WJ and XM did the data collection. ZD did the manuscript correcting and formed the final manuscript. All authors contributed to the article and approved the submitted version.
